# Supplementary material for: Molecular basis for Ras suppressor-1 binding to PINCH-1 in focal adhesion assembly
Source: J Biol Chem. 2021 Apr 21;296:100685. doi: 10.1016/j.jbc.2021.100685 (PMC8141872; doi:10.1016/j.jbc.2021.100685)
Supplement: Figures S1 to S4 and Table S1 [file mmc1.docx]

**Molecular basis for Ras suppressor-1 binding to PINCH-1 in focal adhesion assembly**

**Koichi Fukuda, Fan Lu, and Jun Qin**

**A list of materials included:**

Figure S1

Figure S2

Figure S3

Figure S4

Table S1

**
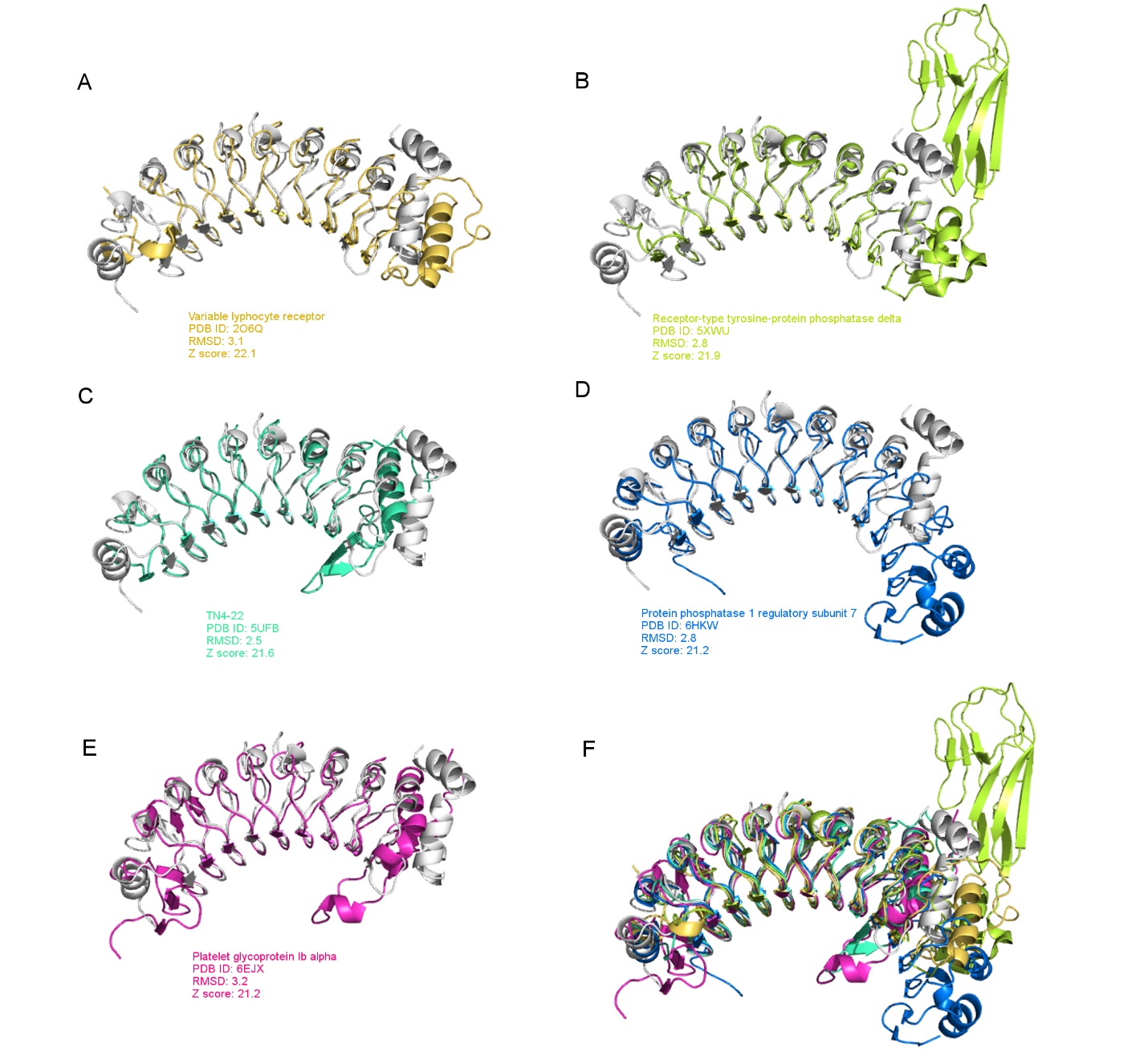
Figure S1. Structural similarity of the LRR domain of Rsu-1 to the selected structural homologues in the PDB90 data set found in the DALI server.** (A-E) Superposition of the structure of the LRR domain of Rsu-1 (white) with the top 5 structural homologues found in the PDB90 dataset. The target molecules were chosen for LRR-containing proteins from animal species. RMSD from the PDB90 dataset is represented in Angstrom (Å) scale. (F) Overlay of all those structural homologues to Rsu-1.

**
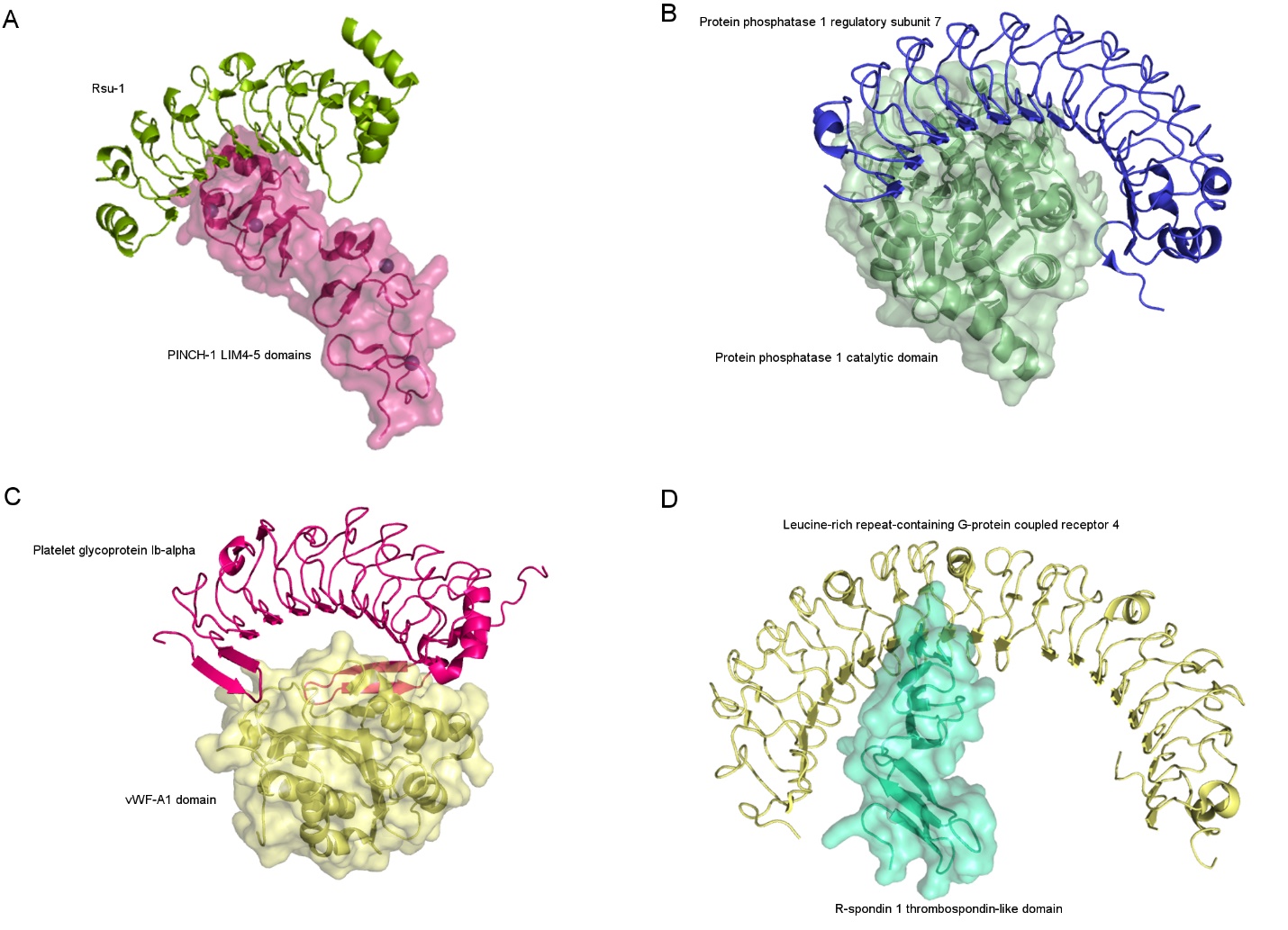
**

**Figure S2. Diversified architectures of LRR-protein ligand complex.** Representative top three coordinates from the PDB90 data set are chosen based on their high Z scores and prototypic LRR-protein ligand complex. (A) The structure of the LRR domain of Rsu-1 (ribbon in green) bound to the zinc-finger type LIM4-5 domains of PINCH-1 (ribbon and transparent surface in magenta). PDB ID: 7LT9 (this study). (B) The structure of the protein phosphatase 1 regulatory subunit 7 (ribbon in blue) bound to the protein phosphatase 1 catalytic domain (ribbon and transparent surface in green). PDB ID: 6OBN. (C) The structure of the platelet glycoprotein Ib-α subunit (ribbon in red) bound to the von Willebrand Factor (vWF) A1 domain (ribbon and transparent surface in yellow). PDB ID: 1M10. (D) The structure of the leucine-rich repeat-containing G-protein coupled receptor 4 (ribbon in yellow) bound to the R-spondin 1 thrombospondin type domain (ribbon and transparent surface in cyan). PDB ID: 4KT1.

**
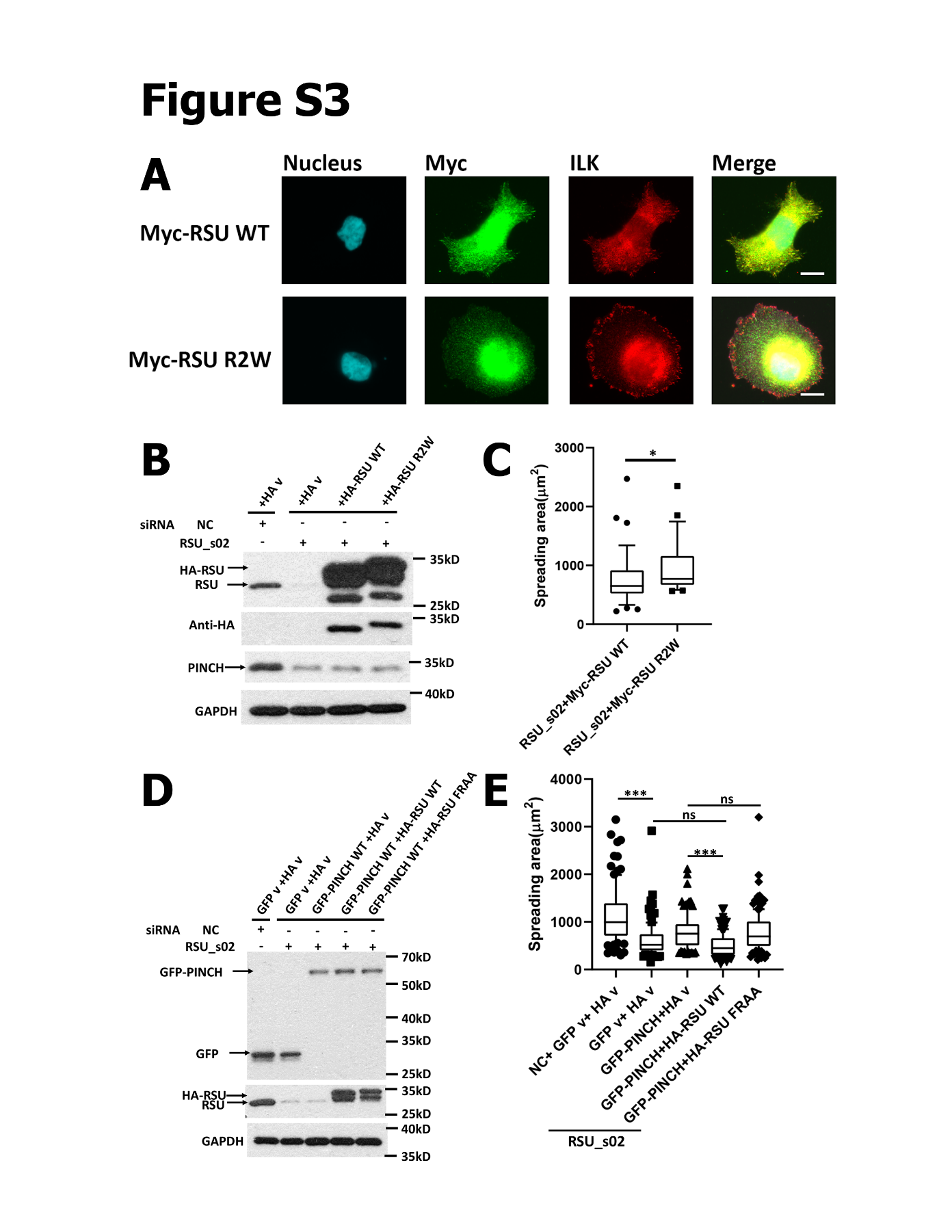
**

**Figure S3**. Rsu-1-mediated regulation on cell spreading. (A) Localization of Myc-tagged Rsu-1 WT and R2W mutant in Rsu-1 siRNA treated MCF10A cells. The Myc-tagged Rsu-1 WT localized to the focal adhesion sites where ILK localized, whereas its R2W mutant did not. Scale bar, 10 µm; (B) Western blot analysis showing Rsu-1 and PINCH-1 levels upon re-expression of Rsu-1 in Rsu-1 siRNA treated MCF10A cells. (C) Quantitative analysis of cell spreading upon expression of Myc-tagged Rsu-1 wild type versus Rsu-1 R2W mutant. Data are plotted as Box and whiskers. (*P=0.011, N=38 or 22) (D) Western blot analysis showing the levels of co-expressed GFP-PINCH-1/HA-Rsu-1 or GFP-PINCH-1/Rsu-1 R2W in PINCH-1/Rsu-1 deficient HeLa cells. (E). Quantitative analysis of cell spreading of HeLa cells upon transfection with GFP-PINCH-1/HA-Rsu-1 or GFP-PINCH-1/Rsu-1 R2W mutant. Data are plotted as Box and whiskers. (***P<0.001, N>100) All Box and Whiskers plots has the center line indicating the median value, while the box contains 25-75 percentile of the dataset. Whiskers mark the 10th and 90th percentile, and value beyond upper and lower bounds are considered as outliers, marked as dots, squares or triangles.

**
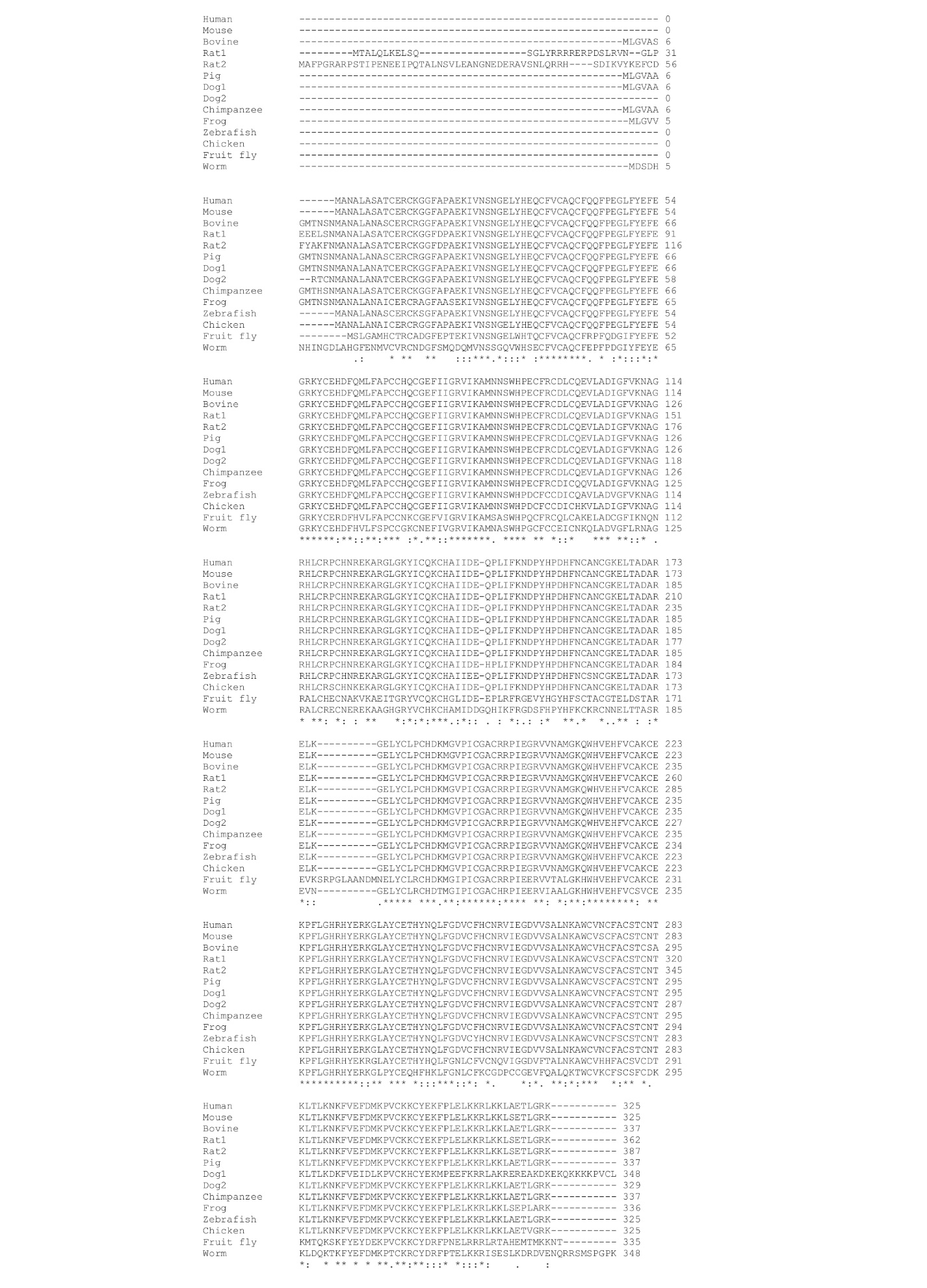
**

**Figure S4. Multiple sequence alignment of PINCH-1 proteins across species.** The sequence alignment was carried out with the program CLUSTAL Omega. As several species contain multiple isoforms of PINCH-1 protein, most representative sequences similar to the human ortholog were presented. Several PINCH-1 isoforms exist in divergent sequences in lengths from some species but those are mostly conserved at the C-terminal regions. It should be noted that dog contains six PINCH-1 isoforms and four out of them exhibit 11-residue extension at the C-terminal region (Dog1 as a representative diversified sequence from NCBI entry A0A5F4CDY4 is included in the multiple sequence alignment). Additional noteworthy is that *Caenorhabditis elegans* PINCH-1 (unc-97) also exhibits 11-residue extension at the C-terminal region. Whether those C-terminal extensions may interfere to the binding to Rsu-1 remains to be determined.

**Table S1. List of the top 20 closest structural homologues in the PDB90 to the LRR domain of Rsu-1 found in the DALI server.** The list is based on the structural similarity score (DALI Z-score). R.m.s.d. stands for the root-mean-square-deviation for the equivalent Cα atom pairs. Nres and %ID represent the total number of residues in the target protein and the sequence identity (percentage), respectively.

| No. | Target protein name | PDB ID | Z | r.m.s.d (Å) | Nres | %ID |
| --- | --- | --- | --- | --- | --- | --- |
| 1 | LIC11098 | 4u08 | 26.6 | 2 | 391 | 33 |
| 2 | LIC10831 | 4u06 | 23.4 | 2.4 | 344 | 36 |
| 3 | OR464 | 4psj | 23.2 | 2.1 | 255 | 32 |
| 4 | Variable lymphocyte receptor | 2o6q | 22.1 | 3.1 | 270 | 29 |
| 5 | LIC12759 | 4u09 | 22.1 | 2 | 396 | 31 |
| 6 | Receptor-type tyrosine-protein phosphatase delta | 5xwu | 21.9 | 2.8 | 343 | 22 |
| 7 | LRR receptor-like serine-threonine protein kinase | 6s6q | 21.8 | 3.4 | 833 | 24 |
| 8 | TN4-22 | 5ufb | 21.6 | 2.5 | 241 | 26 |
| 9 | Probably LRR receptor-like serine-threonine-protein kinase At4g26540 | 5hyx | 21.5 | 3.4 | 626 | 28 |
| 10 | LIC12234 | 4tzh | 21.4 | 1.7 | 190 | 32 |
| 11 | Leucine rich repeat protein | 4r5c | 21.4 | 2.8 | 304 | 30 |
| 12 | Protein TOO MANY MOUTHS | 5xjx | 21.3 | 3.3 | 544 | 26 |
| 13 | Protein phosphatase 1 regulatory subunit 7 | 6hkw | 21.2 | 2.8 | 306 | 27 |
| 14 | platelet glycoprotein Ib α | 6ejx | 21.2 | 3.2 | 266 | 29 |
| 15 | LRRTM | 5a5c | 21.2 | 2.7 | 332 | 30 |
| 16 | Leucine-rich repeat-containing G-protein coupled receptor | 4kt1 | 21.1 | 2.6 | 461 | 27 |
| 17 | O13 | 5uei | 21.1 | 2.7 | 246 | 27 |
| 18 | Uncharacterized protein | 4fcg | 21 | 2.8 | 296 | 24 |
| 19 | Volume-regulated anion channel subunit LRRC8A | 6djb | 20.9 | 2.6 | 725 | 29 |
| 20 | Leucine-rich repeat DLRR_I | 4r6f | 20.8 | 2.6 | 329 | 30 |
